# Projections of utilization of primary and revision shoulder arthroplasty in the United States in the next 40 years

**DOI:** 10.1016/j.jseint.2024.10.013

**Published:** 2024-11-14

**Authors:** Andrew J. Cecora, Dashaun Ragland, Neel Vallurupalli, Erel Ben-Ari, Jacquelyn J. Xu, Brian O. Molokwu, Young W. Kwon, Joseph D. Zuckerman, Mandeep S. Virk

**Affiliations:** Division of Shoulder and Elbow Surgery, Department of Orthopedic Surgery, NYU Grossman School of Medicine, NYU Langone Orthopedic Hospital, NYU Langone Health, New York, NY, USA

**Keywords:** Shoulder arthroplasty, Projection, Utilization, Future, Medicare, Revision arthroplasty

## Abstract

**Background:**

In the past 20 years, the incidence of total shoulder arthroplasty (TSA) has increased greatly, and it is expected to continue growing. Current literature lacks future projections for the utilization of TSA. These projections can help predict demand quantities and anticipate the future burden on the healthcare system. The aim of this study is to determine the predictions of utilization for TSA, primary and revision, through 2060.

**Methods:**

This analysis used the publicly available 2000-2019 data from the Center for Medicare and Medicaid Services Medicare Part-B National Summary. Procedure volumes, including TSA and revision TSA, were determined using Current Procedural Terminology codes and were uplifted to account for the growing number of Medicare eligible patients covered under Medicare Advantage. Log-linear, Poisson, negative binomial regression, and autoregressive integrated moving average models were applied to the procedural volumes to generate projections from 2020-2060. The Poisson model was chosen to display the data based on error analysis and prior literature.

**Results:**

The projected annual growth from 2020 to 2060 rates for primary and revision TSA are 11.65% growth (95% confidence interval 11.60%-11.69%) and 13.89% growth (95% confidence interval 13.35%-14.42%), respectively. By 2060, the demand for primary TSA and revision TSA is projected to be 10,029,260 and 1,690,634, respectively.

**Conclusion:**

The results of this study concluded that both primary and revision TSA procedures are projected to exponentially increase from 2020 to 2060. Additionally, revision procedures are projected to increase at greater rates than their respective primary counterparts.

Total joint arthroplasty (TJA) is one of the most successful and cost-effective musculoskeletal surgical procedures performed in the United States today.^2^^,^^13^^,^^17^ From their inception, TJAs have increased in popularity and indications, among both patients and providers, which is evident by increasing overall volume of procedures.^21^ Current literature shows a continued rise in utilization in the years to come.^15^

The TJA burden is expected to increase in the coming years for many reasons. One reason is that with an aging population, the number of TJAs will likely increase substantially.^14^ Another reason is the documented success in clinical outcomes and the improved longevity of the prostheses. Finally, patients are receiving primary TJAs at earlier ages. Kurtz et al^10^ determined that by 2030, half of all TJA recipients will be aged less than 65 years. With a growing number of young patients receiving TJAs, there will be more revision surgeries performed.^10^

Most published literature focuses on the past trends and future projections of total knee arthroplasty and total hip arthroplasty, which may be because they are more commonly performed than other TJAs.^15^, ^16^, ^17^ There is less literature studying utilization projections for upper extremity arthroplasties such as total shoulder arthroplasty (TSA).^3^^,^^18^ Much of the published literature focuses on current incidence of the procedures, lacking concrete long-term future projections.^4^^,^^5^^,^^8^ One study conducted in France showed utilization projections for TSA until the year 2070.^19^ However, a projection of this length has not been replicated in the United States.

A second study analyzed the prevalence and projections of TSA utilization in the United States.^3^ They analyzed the Nationwide Inpatient Sample from 1993 to 2007 to examine trends in upper extremity arthroplasty and generate projections of utilization up to the year 2015.^3^ One major limitation of this study was the inability to differentiate between primary and revision upper extremity arthroplasty procedure projections because they use the same International Classification of Diseases, 9th Revision, Clinical Modification code.^3^ Currently, revision TSA has a unique Current Procedural Terminology (CPT) codes in the Medicare Part-B database and therefore can have distinct projections created.

Reliable projections can help policy makers, professional societies, hospital administrators, and orthopedic practitioners to set regulations, negotiate contracts, recruit employees, and perform other tasks to ensure delivery of shoulder care, optimize efficiency, and revenue as the market changes.^9^ These projections can help predict demand quantities which will assist policy makers, hospitals, and organizations to anticipate the future burden on the healthcare system and ensure patients receive adequate care. In addition to resource distribution, the lack of current projections for TSA further reinforces the need for an update. To date, no study has included the revision TSA CPT codes for a specific projection. Therefore, the aim of this study is to determine the predictions of utilization for TSA, primary and revision, through 2060. We hypothesize that TSA procedures will increase substantially in the coming decades due to the aforementioned reasons.

## Methods

No institutional review board approval was required for this study.

This analysis used 2000-2019 data from the Center for Medicare and Medicaid Services Medicare Part-B National Summary that has been published and made publicly accessible by Centers for Medicare and Medicaid Services. Procedures including TSA, and revision TSA, were determined using CPT codes. CPT code 23470 and 23472 were used to identify shoulder hemiarthroplasty and TSA (anatomic TSA and reverse TSA), respectively. Revision TSA cases were identified using CPT codes 23473, indicating one component revision (either glenoid or humeral), or 23474 indicating both components were revised (glenoid and humeral).

The dataset used in this study only includes patients enrolling in traditional Medicare. However, there has been a growing proportion of Medicare eligible patients who are covered under Medicare Advantage. As a result, this study used a previously published method of estimating the true prevalence of the Medicare eligible patients by applying a ratio of traditional Medicare to Medicare Advantage patients determined by the Kaiser Family Foundation.^15^ This ratio was applied to the dataset to calculate an uplifted annual volume of primary procedures from the years 2000 to 2019. Data collection for revision procedures began in 2013, as this was when the revision specific CPT codes were introduced.

Like Shichman et al,^15^ we then used the uplifted procedure volumes from 2000 to 2019 and applied log-linear (exponential growth), Poisson, negative binomial regression, and autoregressive integrated moving average models. We used these 4 models to generate time series forecasts with point forecasts and 95% forecast intervals (FIs) for each year between 2020 and 2060. The mean absolute error and root mean square error were determined for each model. Like Day et al,^3^ we chose the Poisson model to show our projections. The Poisson model was chosen because of the lowest standard error when comparing lines of best fit, the lowest or second lowest root mean square error and mean absolute error, and the use of the model in past literature. We then used the forecasted procedure counts for TSA (both anatomic and reverse) and revision TSA to generate projected yearly and 5-year period growth rates. The predictions for volumes of TSA and revision TSA were forecasted and noted over 5-year intervals between 2020 and 2060. This model replicates similar forecasting methodology used to predict growth of other orthopedic procedure volumes and disease prevalence.^15^^,^^17^^,^^20^

The R programming environment version 4.3.1 (2023; The R Foundation for Statistical Computing, Vienna, Austria) was used to perform statistical analysis. R: a language and environment for statistical computing. Foundation for Statistical Computing, Vienna, Austria. ISBN 3-900,051-07-0, URL http://www.R-project.org/.

## Results

The total number of primary TSA and revision TSA procedures increased through each year analyzed. Primary TSA increased from 12,276 to 110,208 over a 19-year period (2000-2019), representing an increase of approximately 798%. Revision TSA saw an increase of 58%, 4988 to 7872, over a 4-year period (2015-2019). This resulted in annual average growth rates of 12% for TSA and 14% for revision TSA. These results are further elaborated on in Table I.

**Table I:** Total number of primary TSA and revision TSA procedures.

Year (5-year intervals)	Primary TSA			Revision TSA		
	Adjusted volume	% change between each 5-year period	Cumulative growth (base = 2000)	Adjusted volume	% change betweeneach 5-year period	Cumulative growth (base = 2015)
2000	12,276	NA	0.00%	NA	NA	NA
2005	24,495	99.53%	99.53%	NA	NA	NA
2010	42,592	73.88%	246.94%	NA	NA	NA
2015	69,218	62.51%	463.82%	4988	NA	0.00%
2019∗	110,208	59.22%	797.72%	7872	57.81%	57.81%

*TSA*, total shoulder arthroplasty.

∗ 2015-2019 represents a 4-year period.

Based on the data in Table II, the TSA procedure projections performed under Medicare varied. The projected annual growth from 2020 to 2060 rates for primary and revision TSA are 11.65% growth (95% confidence interval 11.60%-11.69%) and 13.89% growth (95% confidence interval 13.35%-14.42%), respectively.

**Table II:** TSA procedure projections.

Surgical procedure	Model used	Intercept	Trend estimate	Standard error	Average: projected annual growth (%)	95% CI (lower): projected annual growth (%)∗	95% CI (upper): projected annual growth (%)∗
Primary TSA	Poisson	−210.806919	0.110159192	0.000201	11.65	11.6	11.69
Revision TSA	Poisson	−253.506105	0.130022679	0.0025496	13.89	13.35	14.42

*TSA*, total shoulder arthroplasty; *CI*, confidence interval.

∗Confidence intervals obtained by normal approximation.

**Figure 1:**
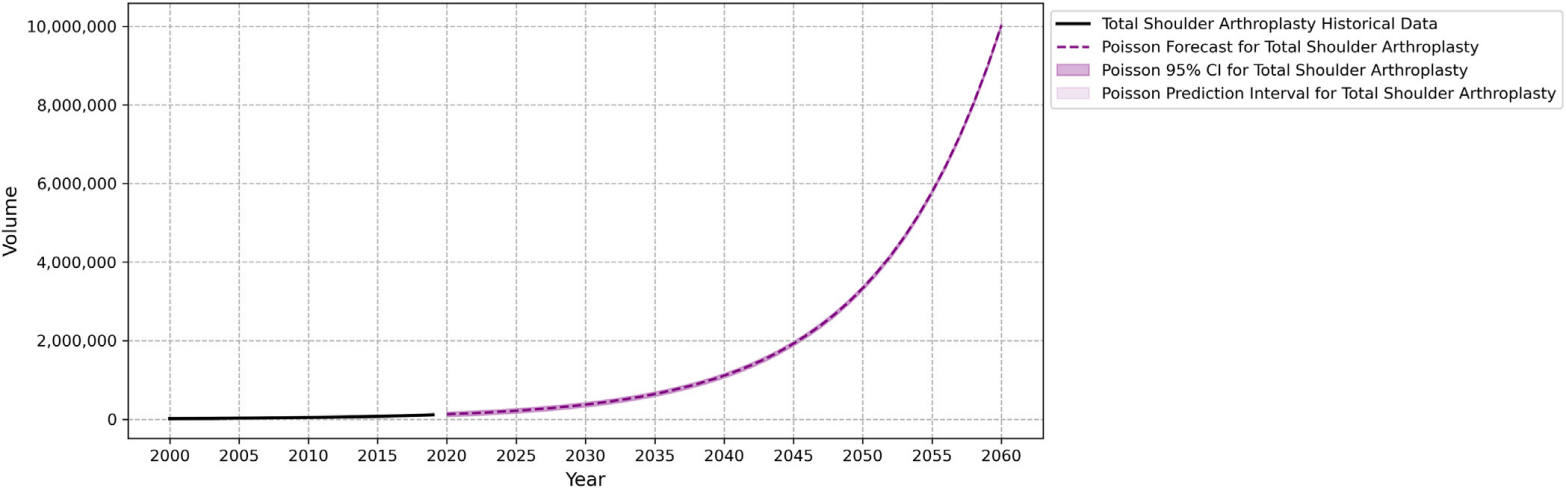
Projected growth of primary TSA. *TSA*, total shoulder arthroplasty; *CI*, confidence interval.

**Figure 2:**
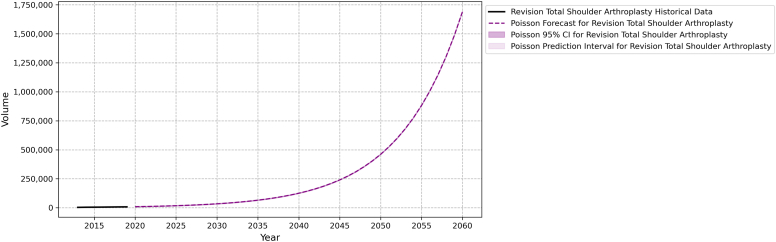
Projected growth of revision TSA. *TSA*, total shoulder arthroplasty; *CI*, confidence interval.

By 2060, the demand for primary TSA and revision TSA is projected to be 10,029,260 (95% FI 10,023,053-10,035,467) and 1,690,634 (95% FI 1,688,086-1,693,183), respectively. The projected cumulative growth rate for primary TSA is 8097.12% and revision TSA is 18,044%. Table III presents a detailed breakdown of 5-year estimates of the primary and revision TSA from 2020 to 2060. The relative differences in projected growth between these 2 procedures can be seen in Figs. 1 and 2.

**Table III:** Breakdown of 5-year projection estimates of primary and revision TSA from 2020-2060.

Year (5-year intervals)	Primary TSA			Revision TSA		
	Projection	95% forecast interval	Cumulative growth (base = 2020)	Projection	95% forecast interval	Cumulative growth (base = 2020)
2020	122,351	121,666-123,037	0.00%	9318	9129-9508	0.00%
2025	212,234	211,332-213,138	73.46%	17,851	17,590-18,113	91.58%
2030	368,148	366,960-369,338	200.90%	34,198	33,836-34,561	267.01%
2035	638,603	637,037-640,169	421.94%	65,516	65,015-66,018	603.11%
2040	1,107,741	1,105,679-1,109,804	805.38%	125,512	124,819-126,207	1246.98%
2045	1,921,525	1,918,808-1,924,242	1470.50%	240,452	239,491-241,413	2480.50%
2050	3,333,140	3,329,562-3,336,719	2624.24%	460,647	459,317-461,978	4843.61%
2055	5,781,776	5,777,063-5,786,489	4625.56%	882,488	880,648-884,330	9370.76%
2060	10,029,260	10,023,053-10,035,467	8097.12%	1,690,634	1,688,086-1,693,183	18,043.68%

*TSA*, total shoulder arthroplasty.

## Discussion

This study was conducted to show predictions of utilization for TSA through the year 2060 in the Medicare population. The results of this study concluded that the primary TSA and revision TSA procedures are projected to exponentially increase from 2020 to 2060. Additionally, revision procedures are projected to increase at greater rates than their respective primary counterparts. The procedure with the greatest projected cumulative growth in prevalence among Medicare patients is revision TSA.

The results of this study mirror the findings noted by Day et al^3^ in their study. Both studies report an increasing burden of revision arthroplasty and increasing utilization of primary shoulder arthroplasty. However, we were able to create projections for revision shoulder arthroplasty as a distinct category, allowing for increased specificity in our updated results. Both Day et al^3^ and our study found that the projection of revision procedures grew at faster rate than did the primary operation. The volume of projected TSA in our study is much greater than the projected volume reported by Day et al,^3^ but this is reflective of the growth of upper extremity arthroplasty procedures from 2007 to 2019. Projection studies using the same modeling algorithms for total hip/knee arthroplasties by Singh et al^16^ and Shichman et al^15^ showed results similar to ours as well, although Shichman et al^15^ used a solely Medicare population, while Singh et al^16^ used 14 years of the national inpatient sample database.

There are several proposed reasons for the exponential increase in TSA prevalence. Increasing exposure to shoulder arthroplasty during residency and fellowship training, increasing comfort level for performing shoulder replacement by orthopedic surgeons from different subspecialties (hand surgery, sports medicine, trauma, and shoulder and elbow surgery), introduction of reverse shoulder replacement, and expanding indications of reverse total shoulder replacement are possible reasons for this exponential increase.^12^ The introduction of reverse TSA as a reliable technique for revision surgery has further increased the utilization.^8^ Increasing prevalence of primary TSA, the aging US population, and growing technological advances in revision shoulder arthroplasty design and instrumentation are contributing to increased utilization of revision TSA.^10^^,^^12^ Padegimas et al^12^ predicted that by 2030, the incidence of shoulder arthroplasty will increase 755% in patients aged more than 55 years, and by 333% in patients aged less than 55 years. Partially attributed to better outcomes for younger patients compared to traditional hemiarthroplasty, this increase in incidence in younger patients further explains the projected increase in revision cases as younger patients are at greater risk for needing revision surgery.^12^

Projecting the future growth of primary and revision TSA is important for multiple reasons. First, it provides a more realistic picture of the disease burden and potential economic impact that the growing disease burden will have in coming decades. Second, these data provide policy makers insight into future need for addressing issues with increasing incidence of revision shoulder arthroplasty. This will require appropriate resource allocation for increasing the surgeon workforce trained for dealing with revision surgery and improving current technologies. Existing data show most primary TSAs are performed by nonshoulder fellowship-trained surgeons,^7^ displaying an unmet need for fellowship-trained shoulder and elbow surgeons specializing in revision arthroplasty. Shoulder and elbow surgery is one of the newest subspecialties with less than 30 years of existence, while shoulder arthroplasty is one of the fastest growing arthroplasty procedures in the United States.^1^^,^^6^^,^^11^ To develop high-volume upper extremity arthroplasty practices to help fill this looming gap in care, efforts will have to be put in place by the American Shoulder and Elbow Surgeons society and the American Academy of Orthopaedic Surgeons to increase fellowship spots and encourage orthopedic residents to pursue subspecialty training in shoulder and elbow surgery or receive more direct education during residency.

As with all studies, our study is not without its limitations. First, we only have 7 years of data for the CPT codes 23473 and 23474 which represent a portion of the revision TSA cohort. These CPT codes were introduced in 2013 to increase specificity of billing procedures for revision TSA because 23473 is for a revision with replacement of a single component, whereas 23474 is used when a revision surgery replaces 2 components. The smaller sample size of 7 years, as opposed to 20 years, impacts the reliability of these projections, and highlights concerns in precision of the coding used prior to 2013. This also is a strength of our article as the lack of these codes completely diminished previous projections validity.^12^ Similarly, there is only 7 years of data for CPT codes 24370 and 24371 because these codes were also introduced in 2013. Second, our study is based off historical Medicare data to create projections; thus, our results are limited in generalizability. Furthermore, these projections may be impacted in the future by improvements in orthopedic implant technology which can improve prosthesis survivorship and therefore reduce the prevalence of revision TSA. On the contrary, a novel nonsurgical treatment for osteoarthritis may considerably reduce the need for primary TSA in the future, as this is what happened to total elbow arthroplasty after the introduction of biologics for rheumatoid arthritis. Unfortunately, there is no way to accurately determine the future impact of technological advances and changes to disease burden. Finally, we chose to create the projections with data ending in 2019 instead of the available 2020 and 2021 data because of the impact of COVID-19 pandemic on surgical volume.

## Conclusion

The results of this study conclude that both primary and revision TSA procedures are projected to exponentially increase from 2020 to 2060. Additionally, revision procedures are projected to increase at greater rates than their respective primary counterparts.

## Disclaimers:

Funding: No funding was disclosed by the authors.

Conflicts of interest: Young W Kwon is a paid consultant for DJO Surgical. Joseph D Zuckerman owns stocks in Apos Therapy, Inc. and Hip Innovation Technology; is a board member of Musculoskeletal Transplant Foundation; received publishing royalties from SLACK Inc., Thieme Inc., and Wolters Kluwer Health; is a Design Surgeon in and receives royalties from Exactech. Mandeep S Virk is a paid consultant for Exactech, Inc. The other authors, their immediate families, and any research foundation with which they are affiliated have not received any financial payments or other benefits from any commercial entity related to the subject of this article.
